# Development and Prospective Validation of an Ultrasound Prediction Model for the Differential Diagnosis of Benign and Malignant Subpleural Pulmonary Lesions: A Large Ambispective Cohort Study

**DOI:** 10.3389/fonc.2021.656060

**Published:** 2021-05-05

**Authors:** Ke Bi, De-meng Xia, Lin Fan, Xiao-fei Ye, Yi Zhang, Meng-jun Shen, Hong-wei Chen, Yang Cong, Hui-ming Zhu, Chun-hong Tang, Jing Yuan, Yin Wang

**Affiliations:** ^1^ Department of Ultrasound, Shanghai Pulmonary Hospital, Tongji University School of Medicine, Shanghai, China; ^2^ Department of Pathology, Tongji Hospital, Tongji University School of Medicine, Shanghai, China; ^3^ Department of Emergency, Changhai Hospital, The Naval Medical University, Shanghai, China; ^4^ Department of Orthopaedics, The Naval Hospital of Eastern Theater Command of People’s Liberation Army of China (PLA), Zhoushan, China; ^5^ Shanghai Clinic and Research Center of Tuberculosis, Department of Tuberculosis, Shanghai Key Laboratory of Tuberculosis, Shanghai Pulmonary Hospital, Tongji University School of Medicine, Shanghai, China; ^6^ Department of Health Statistics, The Naval Medical University, Shanghai, China

**Keywords:** lung ultrasound, contrast-enhanced ultrasound, pulmonary lesion, differential diagnosis, prediction model

## Abstract

**Objective:**

To develop and prospective validate an ultrasound (US) prediction model to differentiate between benign and malignant subpleural pulmonary lesions (SPLs).

**Methods:**

This study was conducted retrospectively from July 2017 to December 2018 (development cohort [DC], n = 592) and prospectively from January to April 2019 (validation cohort [VC], n = 220). A total of 18 parameters of B-mode US and contrast-enhanced US (CEUS) were acquired. Based on the DC, a model was developed using binary logistic regression. Then its discrimination and calibration were verified internally in the DC and externally in the VC, and its diagnostic performance was compared with those of the existing US diagnostic criteria in the two cohorts. The reference criteria were from the comprehensive diagnosis of clinical-radiological-pathological made by two senior respiratory physicians.

**Results:**

The model was eventually constructed with 6 parameters: the angle between lesion border and thoracic wall, basic intensity, lung-lesion arrival time difference, ratio of arrival time difference, vascular sign, and non-enhancing region type. In both internal and external validation, the model provided excellent discrimination of benign and malignant SPLs (C-statistic: 0.974 and 0.980 respectively), which is higher than that of “lesion-lung AT difference ≥ 2.5 s” (C-statistic: 0.842 and 0.777 respectively, *P <*0.001) and “AT ≥ 10 s” (C-statistic: 0.688 and 0.641 respectively, *P <*0.001) and the calibration curves of the model showed good agreement between actual and predictive malignancy probabilities. As for the diagnosis performance, the sensitivity and specificity of the model [sensitivity: 94.82% (DC) and 92.86% (VC); specificity: 92.42% (DC) and 92.59% (VC)] were higher than those of “lesion-lung AT difference ≥ 2.5 s” [sensitivity: 88.11% (DC) and 80.36% (VC); specificity: 80.30% (DC) and 75.00% (VC)] and “AT ≥ 10 s” [sensitivity: 64.94% (DC) and 61.61% (VC); specificity: 72.73% (DC) and 66.67% (VC)].

**Conclusion:**

The prediction model integrating multiple parameters of B-mode US and CEUS can accurately predict the malignancy probability, so as to effectively differentiate between benign and malignant SPLs, and has better diagnostic performance than the existing US diagnostic criteria.

**Clinical Trial Registration:**

www.chictr.org.cn, identifier ChiCTR1800019828.

## Introduction

It is of great importance to differentiate benign and malignant pulmonary lesions since lung cancer is one of the neoplasms with the highest morbidity and mortality in the world (1, 2). Computed tomography (CT), the first-line diagnostic method for lung disease, may fails to diagnose some atypical lesions immediately (3). A definitive diagnosis often requires long-term follow-up or invasive diagnostics, which leads to increased radiation exposure and a high risk of severe complications (4, 5). Given these problems, new noninvasive methods are being explored to enable more precise diagnosis.

Compared with CT, ultrasound (US) has the advantage of real time, non-radiation, and bedside availability, and has been used in assessing lung disease since the 19th century (6). To date, both B-mode US (B-US) and contrast-enhanced US (CEUS) have been proven to be valuable in the differential diagnosis of subpleural pulmonary lesions (SPLs) (7–9). Reportedly, qualitative parameters such as degree and homogeneity of enhancement, perfusion pattern, and vascular sign; and quantitative parameters such as arrival time (AT), lesion-lung AT difference, wash-in rate (WIR) and wash-out rate (WOR) were the potentially useful parameters (8–19). Among them, AT is the most frequently assessed parameter and is recommended by the guidelines proposed by European Federation of Societies for Ultrasound in Medicine and Biology (EFSUMB) (8). It can differentiate benign and malignant SPLs by the difference in the time taken for US contrast agent (UCA) to arrive at the lesion from injection (10–14). However, this parameter is affected by multiple physiological and external factors, such as the length of vessels, cardiac function, and the injection velocity of UCA (20). Therefore, lesion-lung AT difference is proposed, which can exclude the influence of individual factors to a certain extent, and provides better diagnostic performance (13).

Multiple studies have confirmed the effectiveness of US in the diagnosis of SPLs. In B-US images, wedge-shaped, ill-defined margins and aerated bronchus signs are considered as the characteristics of benign lesions, while malignant lesions tended to present spherical and well-defined margins. As for CEUS, AT >7.5s and lesion-lung AT difference >2.5s are recommended as the criteria for predicting malignancy. Nevertheless, they are still limited to the exploration of small samples or single indicators, and the level of evidence is low (8–19). Therefore, the value of lung US requires a larger and more robust study design (9).

We aimed to conduct a large cohort study to explore more valuable parameters of B-US and CEUS and to construct a model for differentiating benign and malignant SPLs with improved accuracy.

## Materials and Methods

This retrospective and prospective cohort study was in accordance with the ethical standards formulated in the Helsinki Declaration and approved by the Institutional Review Board (No. K18-197Y). Moreover, our study protocol followed the statement of Transparent Reporting of a Multivariable Prediction Model for Individual Prognosis or Diagnosis (TRIPOD) and registered in the Chinese Clinical Trial Registry (No. ChiCTR1800019828). All patients signed written informed consent for CEUS, and the patients recruited prospectively also signed separate written informed consent to participate in this clinical trial.

### Patients

We retrospectively collected the medical data of consecutive patients who underwent both B-US and CEUS of lung between July 2017 and December 2018 in a leading pulmonary hospital, and used it as a development cohort (DC) for model establishment and internal validation. Then, we prospectively collected same patient data from January to April 2019 as an external validation cohort (VC) for the model.

The inclusion criteria were (a) the lesion was found for the first time and localized beneath the pleura, using X-ray, CT or US; (b) the lesion was not blocked by artifacts of gas or bones and can be clearly imaged by US. The exclusion criteria were (a) hypersensitivity reaction to UCA (SonoVue; Bracco SpA, Milan, Italy); (b) severe cardiovascular disease; (c) a definite diagnosis had not been successful.

### Image Acquisition and Analysis

**Figure 1:**
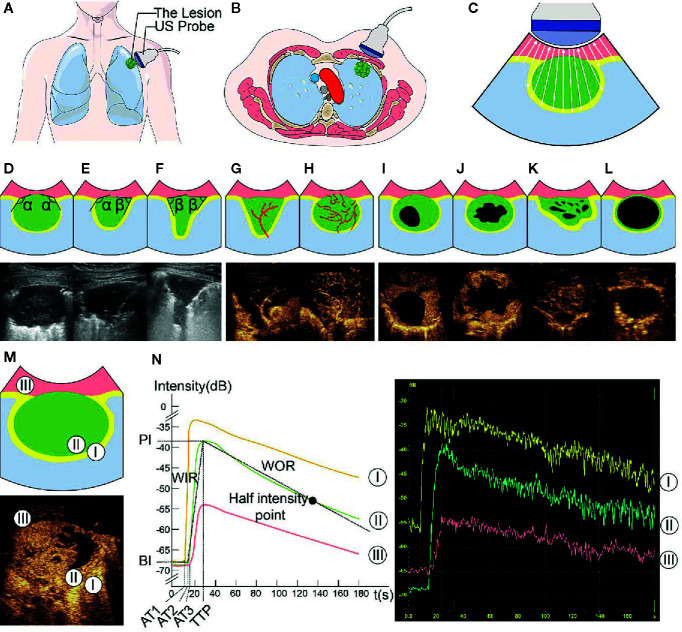
Ultrasound (US) images and parameters of subpleural pulmonary lesions. In this series of figures, the diagrams of the patterns and corresponding US images are displayed in pairs (except for **A–C**). In the diagrams of the patterns, pink represents the thoracic wall, green represents the lesion, yellow represents the enhanced area of air-filled lung tissues in contrast-enhanced US (CEUS) mode, light blue represents the non-enhancing air-filled lung tissues, black represents the non-enhancing region of a lesion, white arrow (in **C**) represents the emission and reception of the US signal, and red line represents the vascular pattern. **(A–C)** Diagrams of patterns show the processes of localization and imaging of the subpleural lung lesion. **(D–F)** In the upper diagrams and lower B-mode US (B-US) images, α represents an obtuse angle between lesion border and thoracic wall and β represents an acute angle. **(G, H)** Upper diagrams of patterns and lower CEUS images show tree-like **(G)** and curly hair-like **(H)** vascular signs. **(I–L)** The non-enhancing regions in the upper diagrams and lower CEUS images are regular, irregular, sieve-like, and almost no enhancement, respectively. **(M)** In the upper diagram of the lesions pattern and lower CEUS image, the numbers 1-3 mark the regions of interest in air-filled lung tissues, lesion, and thoracic wall, respectively. **(N)** The left diagram and the range of instrument outputs (right image) of the time-intensity curves show that the intensities of air-filled lung tissues I, lesion II, and thoracic wall III change with time; the quantitative CEUS parameters are marked on the curves. BI, basic intensity of lesion; AT_1_, arrival time at air-filled lung tissues; AT_2_, arrival time at lesion; AT_3_, arrival time at thoracic wall; TTP, time to peak; PI, peak intensity; WIR, wash-in rate; WOR, wash-out rate.

The process of image acquisition and analysis was unified in the DC and VC. Two radiologists performed this process together (K.B. and Y.Z., with 4 and 5 years of experience in lung US, respectively) using the LOGIQ E9 US System (General Electric Healthcare, Chicago, IL, USA) configured with a 1-6 MHz convex probe. For discordant assessments, a third senior radiologist (Y.W., with 18 years of experience of lung US) was consulted on the cases and would make the final decision. All radiologists had received standardized training before participating in this study. Radiologists were required to locate the probe parallel to the intercostal space and on the largest section of the lesion to obtain B-US and CUES images, and once the probe is positioned, it cannot be moved. The specific method of image acquisition and analysis and the definition of US parameters are given in **Figure 1** and **Table 1**.

**Table 1:** Definitions of Ultrasound Parameters.

US Parameters	Definition	Recorded content
Transverse diameter	The maximum diameter of the lesion parallel to the thoracic wall.	value, cm
Longitudinal diameter	The maximum diameter of the lesion perpendicular to the thoracic wall.	value, cm
Angle between lesion border and thoracic wall	As long as one angle is obtuse, the parameter was classified as obtuse.	obtuse or acute
Perfusion pattern	The way UCA enters the lesion. For a lesion manifesting overlapping patterns, the one with the widest involvement is recorded.	hilum-to-pleura, periphery-to-center or part -to-whole
Degree of enhancement	The enhanced degree of air-filled lung tissues is defined as hyper-enhancement and the enhanced degree of thoracic wall muscle is defined as hypo-enhancement.	hyper-, iso- or hypo- enhancement
Homogeneity	Distribution uniformity of UCA in the lesion.	homogeneous or heterogeneous
Vascular sign	The morphological character of the earliest enhanced blood vessels in the lesion.	negative, tree-like or curly hair-like
Non-enhancing region type	The morphological characteristic of non-enhancing region.	negative, regular, irregular, sieve-like, or almost no enhancement
AT of lung tissue	The time taken for UCA to arrive at air-filled lung tissues from injection	value, s
AT of thoracic wall	The time taken for UCA to arrive at thoracic wall from injection	value, s
AT of lesion	The time taken for UCA to arrive at the lesion from injection	value, s
Lesion-lung AT difference	The AT difference between lesion and air-filled lung tissues.	value, s
Ratio of AT difference	The ratio of “AT difference between lesion and air-filled lung tissues” to “AT difference between thoracic wall and air-filled lung tissues”.	value, %
BI	Initial enhancement intensity.	value, dB
PI	Maximum enhancement intensity.	value, dB
TTP	The time taken by UCA from injection to PI.	value, s
WIR	The growth rate of the intensity from base to peak.	value, dB/s
WOR	The attenuation rate of the intensity from peak to half.	value, dB/s

US, ultrasound; UCA, ultrasound contrast agent; AT, arrival time; BI, basic intensity; PI, peak intensity; TTP, time to peak; WIR, wash-in rate; WOR, wash-out rate.

B-mode US (B-US) parameters were recorded in the largest section of the lesion with general instrument settings: (1) transverse diameter, (2) longitudinal diameter, and (3) the angle between lesion border and thoracic wall.

In contrast-enhanced mode, the mechanical index was set at 0.1, and the gain was adjusted to show the surface of air-filled lungs only (20 dB). Then 1.5 ml of UCA was injected into the median cubital vein within 2s *via* a 20-gauge needle, followed by an immediate flush with 5 mL of normal saline and the dynamic clip was recorded for 3 minutes (8, 20).

Qualitative CEUS parameters were obtained by observing dynamic clips frame by frame: (a) perfusion pattern, (b) degree of enhancement, (c) homogeneity, (d) vascular sign, and (e) non-enhancing region type.

Quantitative CEUS parameters were measured and calculated according to the time-intensity curve (TIC). We used the TIC analysis software in LOGIQ E9 to select the earliest enhancing areas of air-filled lung tissues, the thoracic wall and the lesion as the regions of interest (ROIs) to plot TICs and obtained the following parameters: (a) AT of lung, (b) AT of thoracic wall, (c) AT of lesion, (d) lesion-lung AT difference, (e) ratio of AT difference, (f) basic intensity (BI), (g) peak intensity (PI), (h) time to peak (TTP), (i) WIR, (j) WOR (9).

### Reference Standards

The diagnosis of all lesions was made by two senior respiratory physicians. Histopathology is in priority, when histopathology could not make a definite diagnosis, microbial evidence, imaging findings, clinical symptoms, and treatment effects were required to make a comprehensive analysis. In addition, all cases were followed up for at least 12 months. Respiratory physicians, Pathologists, laboratorian and patients were blinded to our image analysis results.

### Statistical Analyses

SPSS V.20.0 (SPSS Inc., Chicago, IL, USA) and R software V.3.6.0 (Institute for Statistics and Mathematics, Vienna, VIC, Austria) were used for statistical analysis. Categorical data were expressed as absolute numbers and percentages, while continuous data were shown as medians and interquartile range (IQR) or mean ± standard deviation. *P* values of <0.05 were considered to reflect statistical significance.

The prediction model was developed based on the data of the DC. A univariate analysis was firstly performed to select US parameters showing significant differences between benign and malignant lesions as candidate variables. Then, a multivariate analysis using binary logistic regression (forward stepwise method) was performed to screen the candidate variables and establish a prediction model. Finally, the model was used to calculate the malignancy probability of each case to plot the ROC curve and determine the cutoff value when Youden’s index reached its maximum.

The data of the DC and VC were used for internal and external validation of the model respectively. The C-statistic was used to assess the discrimination and the calibration curve based on 1000 bootstrap re-samples was used to evaluate the agreement between actual and predictive malignancy probabilities. Finally, the diagnostic ability of the model and the existing US diagnostic criteria for malignant lesions: “lesion-lung AT difference ≥ 2.5 s” (13) and “AT ≥ 10 s” (14) was compared.

## Results

### Patient Demographic and Clinical Characteristics

**Figure 2:**
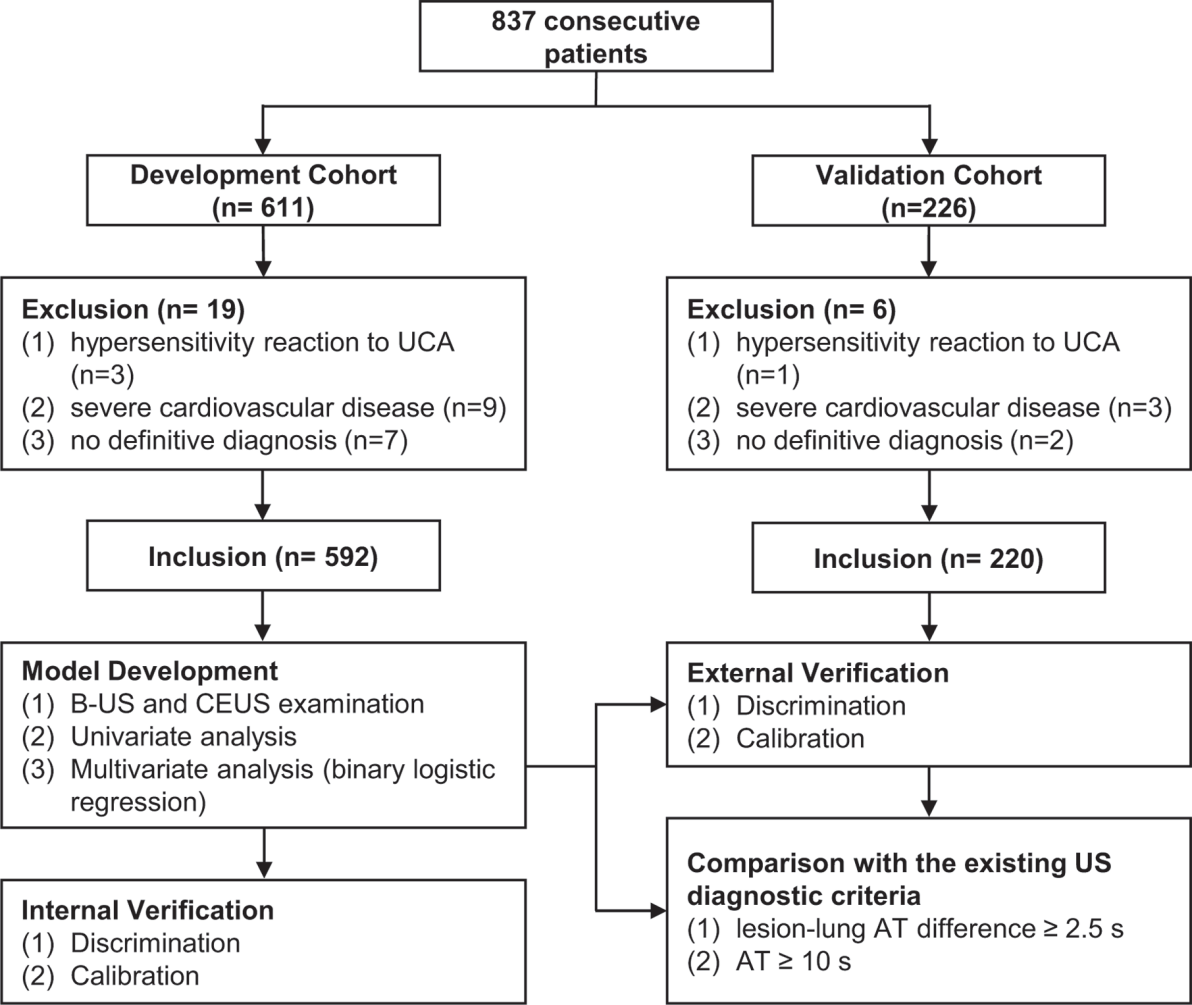
Participant selection and research process. UCA, ultrasound contrast agent; US, ultrasound; B-US, B-mode ultrasound; CEUS, contrast-enhanced ultrasound.

A total of 837 consecutive patients were enrolled in our study, including 592 in the DC and 220 in the VC; while 19 and 6 patients were excluded from each respective cohort (**Figure 2**). The demographic and clinical characteristics of both cohorts were comparable (**Table 2**).

**Table 2:** Patient Demographic and Clinical Characteristics.

Characteristics	Development Cohort (n=592)			Validation Cohort (n=220)		
	Malignant group	Benign group	*P* value	Malignant group	Benign group	*P* value
No. of cases (n, %)	328 (55.41)	264 (44.59)		112 (50.91)	108 (49.09)	
Age, years (M, IQR)	66 (60, 72)	51 (34, 63)	<0.001^a^	65 (58-71)	54 (37.5-67.25)	<0.001 ^a^
Gender (n, %)			<0.001 ^b^			0.015 ^b^
Male	264 (80.49)	164 (62.12)		89 (79.46)	70 (64.81)	
Female	64 (19.51)	100 (37.88)		23 (20.54)	38 (35.19)	
Location (n, %)			0.719 ^b^			0.153 ^b^
Left	143 (43.60)	119 (45.08)		50 (44.64)	38 (35.19)	
Right	185 (56.40)	145 (54.92)		62 (55.36)	70 (64.81)	
Definitive diagnosis (n, %)						
	LUAD, 162 (49.39)	Pneumonia, 111 (42.05)		LUAD, 57 (50.89)	Pneumonia, 46 (42.59)	
	LUSC, 128 (39.02)	TB, 118 (44.70)		LUSC, 43 (38.39)	TB, 39 (36.11)	
	SCLC, 25 (7.62)	NTM, 8 (3.03)		SCLC, 6 (5.36)	NTM, 3 (2.78)	
	LCLC, 1 (0.30)	Fungal, 8 (3.03)		SC, 1 (0.89)	Fungal, 5 (4.63)	
	PDC, 8 (2.44)	Abscess, 7 (2.65)		PDC, 4 (3.57)	Abscess, 5 (4.63)	
	PEComa, 1 (0.30)	ILD, 12 (4.55)		PEComa, 1 (0.89)	ILD, 10 (9.26)	

LUAD, lung adenocarcinoma; LUSC, lung squamous cell carcinoma; SCLC, small cell lung cancer; LCLC, large cell lung cancer; PDC, poorly differentiated carcinoma; PEComa, perivascular epithelioid cell tumors; SC, sarcomatoid carcinoma; TB, tuberculosis; NTM, nontuberculosis mycobacteria; ILD, interstitial lung disease.

M, median; IQR, interquartile range.

a Wilcoxon rank-sum test.

b χ^2^ test.

### Univariate Analysis of US Parameters

Using data from the DC, 14 out of 18 US parameters showed statistical differences between benign and malignant lesions and were selected as candidate variables. The details are as follows (**Table 3**).

**Table 3:** Univariate Analysis of Ultrasound Parameters.

Ultrasound Parameters	Development Cohort (n=592)			Validation Cohort (n=220)		
	Malignant group	Benign group	*P* value	Malignant group	Benign group	*P* value
Transverse diameter, cm (M, IQR)	5.46 (3.83-7.97)	3.88 (2.76-5.41)	<0.001^a^	4.65 (3.49-6.83)	4.26 (3.13-5.32)	0.010^a^
Longitudinal diameter, cm (M, IQR)	4.33 (3.12-5.88)	3 (2.18-4.24)	<0.001^a^	3.93 (2.91-5.59)	3.24 (2.51-4.32)	0.001^a^
Angle between lesion border and thoracic wall (n, %)			<0.001^b^			<0.001^b^
Obtuse	302 (92.07)	131 (49.62)		105 (93.75)	47 (43.52)	
Acute	26 (7.93)	133 (50.38)		7 (6.25)	61 (56.48)	
Perfusion pattern (n, %)			<0.001^c^			<0.001^c^
Hilum-to-pleura	104 (31.71)	211 (79.92)		43 (38.39)	88 (81.48)	
Periphery-to-center	198 (60.37)	42 (15.91)		63 (56.25)	15 (13.89)	
Part-to-whole	26 (7.93)	11 (4.17)		6 (5.36)	5 (4.63)	
Degree of enhancement (n, %)			0.041^d^			0.151 ^d^
Hyper-enhancement	145 (44.21)	133 (50.38)		53 (47.32)	44 (40.74)	
Iso-enhancement	114 (34.76)	95 (35.98)		44 (39.29)	40 (37.04)	
Hypo-enhancement	69 (21.04)	36 (13.64)		15 (13.39)	24 (22.22)	
Homogeneity (n, %)			0.120^b^			0.051^b^
Homogeneous	76 (23.17)	76 (28.79)		18 (16.07)	29 (26.85)	
Heterogeneous	252 (76.83)	188 (71.21)		94 (83.93)	79 (73.15)	
Vascular sign (n, %)			<0.001^c^			<0.001^c^
Neg	242 (73.78)	187 (70.83)		81 (72.32)	77 (71.30)	
Tree-like	18 (5.49)	73 (27.65)		4 (3.57)	29 (26.85)	
Curly hair-like	68 (20.73)	4 (1.52)		27(24.11)	2 (1.85)	
Non-enhancing region type (n, %)			<0.001^c^			<0.001^c^
Neg	149 (45.43)	128 (48.49)		49 (43.75)	48 (42.86)	
Regular	27 (8.23)	45 (17.05)		6 (5.36)	24 (21.43)	
Irregular	137 (41.77)	21(7.95)		54 (48.21)	8 (7.14)	
Sieve-like	8 (2.44)	38 (14.39)		2 (1.79)	15 (13.39)	
Almost no enhancement	7 (2.13)	32 (12.12)		1 (0.89)	13 (11.61)	
AT of lung tissue, s (M, IQR)	6.02 (4.23-8.42)	6.24 (3.79-8.47)	0.596^a^	5.36 (3.74-8.25)	6.5(4.20-9.59)	0.176^a^
AT of thoracic wall, s (M, IQR)	13.46 (10.86-16.55)	12.7 (10.22-15.95)	0.190^a^	12.85 (10.39-16.2)	13.7 (11.38-16.97)	0.089^a^
AT of lesion, s (M, IQR)	11.37 (9.14-14.89)	8.03 (5.36-10.31)	<0.001^a^	11.06 (8.47-14.04)	8.29 (5.62-10.83)	<0.001^a^
Lesion-lung AT difference, s (M, IQR)	5.35 (3.79-6.69)	1.35 (0.78-2.23)	<0.001^a^	4.77 (3.29-7.35)	1.54 (0.69-2.47)	<0.001^a^
Ratio of AT difference, % (M, IQR)	76.87 (62.59-95.45)	23.34 (13.75-33.29)	<0.001^a^	77.54 (62.61-88.5)	20.27 (12.01-30.40)	<0.001^a^
BI, dB (M, IQR)	-67.18 [(-68.08)-(-65.53)]	-65.44 [(-67.15)-(-62.5)]	<0.001^a^	-67.29 [(-68.13)-(-65.65)]	-65.61 [(-66.9)-(-63.48)]	<0.001^a^
PI, dB (M, IQR)	-42.99 ± 6.79	-40.77 ± 6.55	<0.001^e^	-43.95 ± 6.53	-41.58 ± 5.75	0.005^e^
TTP, s (M, IQR)	22.29 (18.52-27.63)	18.5 (13.79-25.59)	<0.001^a^	21.63 (18.14-25.91)	17.76 (12.8-24.16)	<0.001^a^
WIR, dB/s (M, IQR)	2.3 (1.64-3.17)	2.44 (1.51-3.66)	0.547^a^	2.22 (1.66-2.90)	2.42 (1.70-3.60)	0.104^a^
WOR, dB/s (M, IQR)	0.13 (0.1-0.18)	0.09 (0.08-0.13)	<0.001^a^	0.12 (0.09-0.16)	0.09 (0.07-0.12)	<0.001^a^

M, median; IQR, interquartile range; Neg, negative; AT, arrival time; Lesion-lung AT difference, the AT difference between lesion and air-filled lung tissues; Ratio of AT difference, the ratio of “AT difference between lesion and air-filled lung tissues” to “AT difference between thoracic wall and air-filled lung tissues”; BI, basic intensity; TTP, time to peak; PI, peak intensity; WIR, wash-in rate; WOR, wash-out rate.

a Wilcoxon rank-sum test.

b χ^2^ test.

c χ^2^ test and multiple comparisons (Bonferroni) showed significant differences between any 2 groups.

d Kruskal-Wallis test.

e t test.

All of 3 B-US parameters showed significant statistical differences between benign and malignant lesions. The transverse and longitudinal diameters of malignant lesions were larger than those of benign lesions [transverse diameter: 5.46 (3.83-7.97) vs 3.88 (2.76-5.41), *P* <0.001; longitudinal diameter: 4.33 (3.12-5.88) vs 3 (2.18-4.24), *P* <0.001]. The angles between the lesion border and thoracic walls were mainly obtuse in the malignant lesions, while the frequencies of the obtuse and acute angles were similar in the benign lesions [obtuse/acute: 302 (92.07%)/26 (7.93%) vs 131 (49.62%)/133 (50.38%), *P* <0.001].

Among the 5 qualitative CEUS parameters, 4 parameters had significant statistical differences between benign and malignant lesions, which were (1) perfusion pattern (*P* <0.001): hilum-to-pleura (31.71% vs 79.92%), periphery-to-center (60.37% *vs* 15.91%) and part-to-whole (7.93% vs 4.17%); (2) degree of enhancement (*P* = 0.041): hyper-enhancement (44.21% vs 50.38%), iso-enhancement (34.76% vs 35.98%) and hypo-enhancement (21.04% vs 13.64%); (3) vascular sign (*P* < 0.001): tree-like (5.49% vs 27.65%) and curly hair-like (20.73% vs 1.52%); and (4) non-enhancing region type (*P* < 0.001): regular (8.23% vs 17.05%), irregular (41.77% vs 7.95%), sieve-like (2.44% vs 14.39%) and almost no enhancement (2.13% vs 12.12%). Only 1 parameter were not statistically different between benign and malignant lesions. It was homogeneity (*P* = 0.120): homogeneous (23.17% vs 28.79%) and heterogeneous (76.83% vs 71.21%).

In terms of the 10 quantitative CEUS parameters, 7 parameters had significant statistical differences between benign and malignant lesions. They were (1) AT of lesion (11.37s vs 8.03s, *P <*0.001), (2) lesion-lung AT difference (5.35s vs 1.35s, *P <*0.001), (3) ratio of AT difference (76.87% vs 23.34%, *P <*0.001), (4) BI (-67.18dB vs -65.44dB, *P <*0.001), (5) PI (-42.99dB vs -40.77dB, *P <*0.001), (6) TTP (22.29dB vs 18.5dB, *P <*0.001) and (7) WOR (0.13dB/s vs 0.09dB/s, *P <*0.001). There was no difference in the 3 parameters between benign and malignant lesions: (1) AT of lung tissue (6.02s vs 6.24s, *P* =0.596), (2) AT of thoracic wall (13.46s vs 12.7s, *P* =0.19) and (3) WIR (2.3dB/s vs 2.44dB/s, *P* =0.547).

### Multivariate Analysis and Model Establishment

Among the 14 candidate variables, the following 6 were selected for modeling (**Table 4**): the angle between lesion border and thoracic wall (obtuse vs. acute, standardized regression coefficient [*S.β*] -0.506, odds ratio [*OR*] 7.908, *P* <0.001), BI (*S.β* -0.607, *OR* 0.695, *P* < 0.001), lesion-lung AT difference (*S.β* 0.366, *OR* 1.262, *P* = 0.044), ratio of AT difference (*S.β* 1.302, *OR* 1.063, *P* < 0.001), vascular sign (tree-like sign, *S.β* -0.232, *OR* 0.312, *P* = 0.015; curly hair-like sign, *S.β* 0.404, *OR* 9.410, *P* = 0.013), and non-enhancing region type (regular, *S.β* -0.270, *OR* 0.224, *P* = 0.004; irregular, *S.β* 0.004, *OR* 1.019, *P* = 0.972; sieve-like, *S.β* -0.388, *OR* 0.072, *P* = 0.001; and almost no enhancement, *S.β* -0.527, *OR* 0.021, *P* < 0.001).

**Table 4:** Multivariate Analysis of Candidate Variables Derived from the Development Cohort.

Risk factors	*β*	*S.E.*	*S.β.*	*Walds*	*df*	*P* value	*OR* (95% *CI*)
Angle between lesion border and thoracic wall (obtuse, acute angle as reference)	2.068	0.405	-0.506	26.013	1	<0.001	7.908 (3.572-17.506)
BI	-0.364	0.079	-0.607	21.250	1	<0.001	0.695 (0.595-0.811)
Lesion-lung AT difference	0.233	0.116	0.366	4.037	1	0.044	1.262 (1.006-1.583)
Ratio of AT difference	0.061	0.011	1.302	31.586	1	<0.001	1.063 (1.041-1.086)
Vascular sign							
Neg (reference)	–	–	–	13.391	2	0.001	–
Tree-like	-1.166	0.479	-0.232	5.928	1	0.015	0.312 (0.122-0.797)
Curly hair-like	2.242	0.904	0.404	6.147	1	0.013	9.410 (1.599-55.366)
Non-enhancing region type							
Neg (reference)	–	–	–	30.907	4	<0.001	–
Regular	-1.494	0.513	-0.270	8.474	1	0.004	0.224 (0.082-0.614)
Irregular	0.018	0.532	0.004	0.001	1	0.972	1.019 (0.359-2.892)
Sieve-like	-2.626	0.771	-0.388	11.604	1	0.001	0.072 (0.016-0.328)
Almost no enhancement	-3.846	0.948	-0.527	16.475	1	<0.001	0.021 (0.003-0.137)
Constant	-28.208	5.340	–	27.908	1	<0.001	0

β, regression coefficient; S.E., standard error; S.β, standardized regression coefficient; df, degree of freedom; OR, odds ratio; CI, confidence interval; BI, basic intensity; Lesion-lung AT difference, the AT difference between lesion and air-filled lung tissues; Ratio of AT difference, the ratio of “AT difference between lesion and air-filled lung tissues” to “AT difference between thoracic wall and air-filled lung tissues”; Neg, negative.

**Figure 3:**
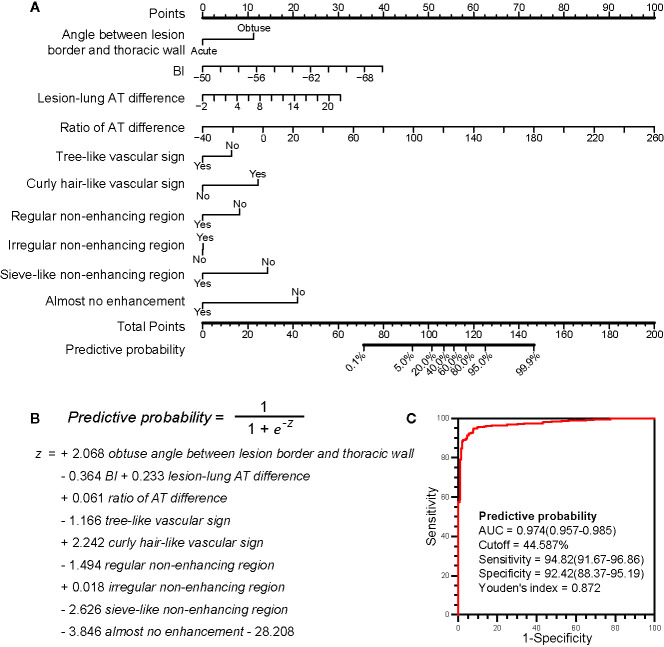
The ultrasound prediction model and its receiver operating characteristic (ROC) curve. **(A)** The model in nomogram form. To use the nomogram, start at the value of each risk factor of an individual lesion located on the corresponding axis. Draw a line up to the horizontal Points axis at the top of the nomogram and record the corresponding points. Locate the sum of these points on the horizontal Total Points axis at the bottom of the nomogram and draw a line further down to the Predictive probability axis to determine the probability. **(B)** The model in formula form. The prediction of malignancy probability can be calculated using the formula. The values of BI, ratio of AT difference and lesion-lung AT difference are directly put into the formula, and the values of other parameters are 1 (Yes) or 0 (No). **(C)** The ROC curve of the model. The larger the area under the curve, the stronger the ability to differentiate between benign and malignant lesions. The best cutoff value and the diagnostic performance are shown in detail. BI, basic intensity; AT, arrival time; Lesion-lung AT difference, the AT difference between lesion and air-filled lung tissues; Ratio of AT difference; the ratio of “AT difference between lesion and air-filled lung tissues” to “AT difference between thoracic wall and air-filled lung tissues”.

The US prediction model is shown in **Figures 3A, B** in the form of nomogram and formula, which can be used to predict the malignancy probability of a lesion. The detailed method of application was explained in the figure legend.

**Figure 4:**
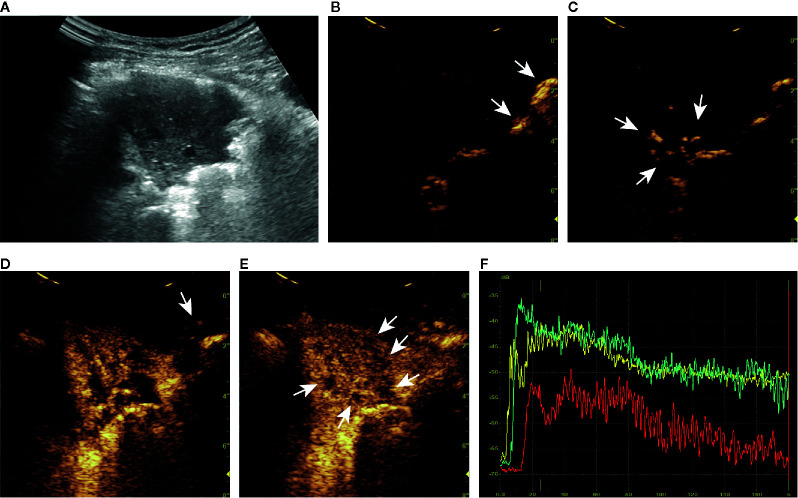
A series of ultrasound images of a benign subpleural pulmonary lesion (tuberculosis) in the upper lobe of left lung of a 61-year-old woman. B-mode ultrasound **(A)** showed the largest section of the lesion, and the angle between lesion border and thoracic wall was acute. The ultrasound contrast agent arrived at air-filled lung tissues (arrow) at about 4 s **(B)**, the lesion (arrow) at 7 s **(C)** and the thoracic wall (arrow) at 14 s **(D)**. At about 15 s, the lesion was completely enhanced and the intensity reached the peak **(E)**. There was a tree-like vascular sign **(D)** and sieve-like non-enhancing regions (arrow) in the lesion **(E)**. Accurate quantitative contrast-enhanced ultrasound parameters were obtained from the time-intensity curves of air-filled lung tissues (yellow curve), the lesion (green curve), and the thoracic wall (red curve) **(F)**: basic intensity = -67.64 dB, lung-lesion arrival time difference = 2.56 s, ratio of arrival time difference = 26.18%. The malignant probability calculated by the ultrasound prediction model was 0.55% < 45.59%, so the lesion was predicted to be benign, which was consistent with the definite diagnosis. The corresponding dynamic clip is shown in **Supplementary Video 1**.

**Figure 5:**
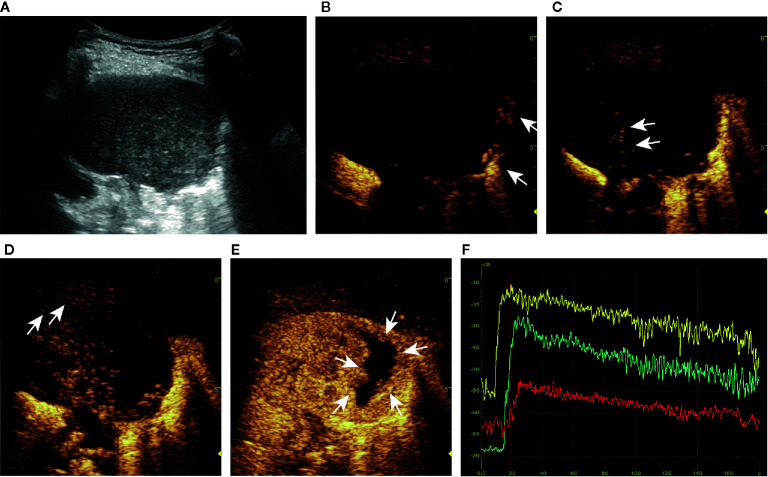
A series of ultrasound images of a malignant subpleural pulmonary lesion (squamous cell carcinoma) in the upper lobe of left lung of a 75-year-old man. B-mode ultrasound **(A)** showed the largest section of the lesion, and the angle between lesion border and thoracic wall was obtuse. The ultrasound contrast agent arrived at air-filled lung tissues (arrow) at about 9 s **(B)**, the lesion (arrow) at 14 s **(C)** and the thoracic wall (arrow) at 18 s **(D)**. At about 24 s, the lesion was completely enhanced and the intensity reached the peak **(E)**. There was no obvious vascular sign in the lesion **(C, D)**, and the non-enhancing region (arrow) was irregular **(E)**. Accurate quantitative CEUS parameters can be obtained from the time-intensity curves of air-filled lung tissues (yellow curve), lesion (green curve), and the thoracic wall (red curve) **(F)**: basic intensity = -68.22 dB, lung-lesion arrival time difference = 6.02 s, ratio of arrival time difference = 65.86%. The malignant probability calculated by the ultrasound prediction model was 98.42% > 45.59%, so the lesion was predicted to be malignant, which was consistent with the definite diagnosis. The corresponding dynamic clip is shown in **Supplementary Video 2**.

The cutoff value obtained from the ROC curve was 44.59%, that was, when the malignancy probability was ≥ 45.59%, the lesion was judged as malignant (**Figure 3C**). The applications of the model in benign and malignant SPLs are shown in **Figure 4** and **Figure 5** (the corresponding dynamic clips are **Supplementary Videos 1, 2**).

### Model Validation

In terms of discrimination, the C-statistics of the model were 0.974 (95% confidence interval [CI]: 0.957-0.985) and 0.980 (95% CI 0.951-0.994) in the internal (DC) and external (VC) verification cohorts, respectively, which is significantly higher than those of “lesion-lung AT difference ≥ 2.5 s” [DC, 0.842 (95% CI 0.810-0.871);VC 0.777 (95% CI 0.716-0.830), *P <*0.001] and “AT ≥ 10 s” [DC, 0.688 (95% CI 0.649-0.725);VC 0.641 (95% CI 0.574-0.705), *P <*0.001].

**Figure 6:**
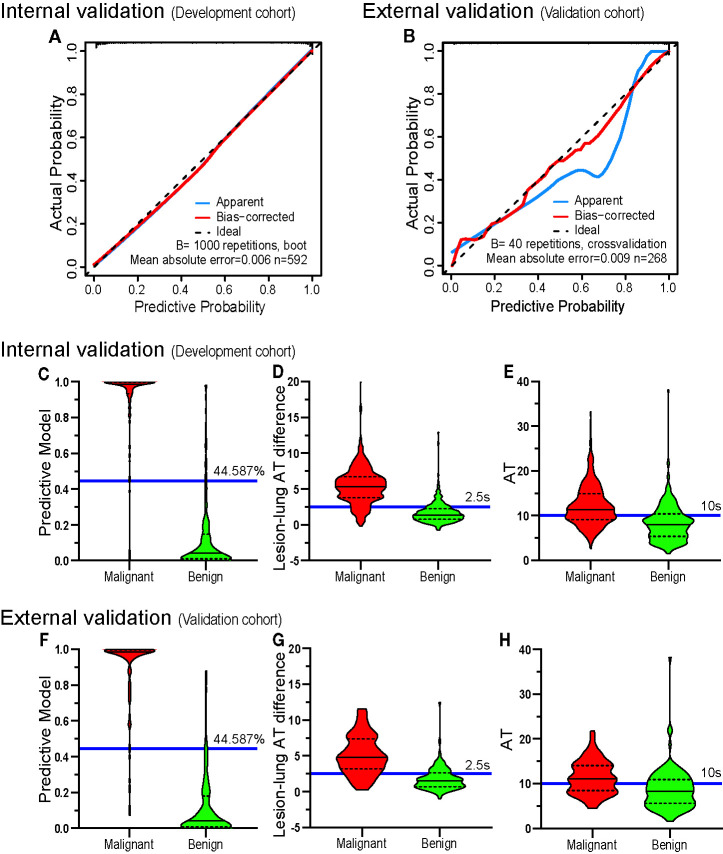
The validation of discrimination and calibration internally and externally. The calibration curves **(A, B)** of the prediction model show good agreement between the actual probabilities and predictive probabilities for the internal verification cohort (DC) and the external verification cohort (VC) (the closer the calibration curve is to the diagonal, the higher the calibration of the model). The diagnostic results of the prediction model **(C, F)**, lesion-lung AT difference **(D, G)** and AT **(E, H)** are depicted by violin plots. The red colored images represent malignant cases, the green-colored images represent benign cases, the horizontal solid lines indicate the median, the dotted lines indicate the quartiles, the blue horizontal line outside the images and the accompanying values are the cutoff values. The graphed data above the blue horizontal line are considered malignant, and the graphed data below the line are considered benign. It can be seen that the discriminatory abilities of the prediction model **(C, F)** are higher than those of the lesion-lung AT difference **(D, G)** and AT **(E, H)**. AT, arrival time; Lesion-lung AT difference, the AT difference between lesion and air-filled lung tissues.

As for calibration, the calibration curves showed good agreement between actual and predictive malignancy probabilities. Although the apparent probability of the external validation cohort showed a slight deviation, the bias-corrected probability improved the final result (**Figures 6A, B**).

### Comparison of the Diagnostic Performance of the Model and the Existing US Diagnostic Criteria

The sensitivity (DC, 94.82%; VC, 92.86%), specificity (DC, 92.42%; VC, 92.59%), positive predictive value (DC, 93.96%; VC, 92.86%), and negative predictive value (DC, 93.49%; VC, 92.59%) of the model were higher than those of the existing US diagnostic criteria: “lesion-lung AT difference ≥ 2.5 s” (sensitivity: DC, 88.11%; VC, 80.36%; specificity: DC, 80.30%; VC, 75.00%; positive predictive value: DC, 84.75%; VC, 76.92%; and negative predictive value: DC, 84.46%; VC, 78.64%), and “AT ≥ 10 s” (sensitivity: DC, 64.94%; VC, 61.61%; specificity: DC, 72.73%; VC, 66.67%; positive predictive value: DC, 74.74%; VC, 65.71%; and negative predictive value: DC, 62.54%; VC, 62.61%). Detailed results are presented in **Figures 6C–H**, and **Table 5**.

**Table 5:** Comparison of Diagnostic Ability.

	Prediction model^a^	Lesion-lung AT difference^b^	AT^c^
**Development Cohort**			
True positive/False negative	311/17	289/39	213/115
False positive/True negative	20/244	52/212	72/192
Sensitivity (%, 95% *CI*)	94.82 (91.67-96.86)	88.11 (83.99-91.31)	64.94 (59.47-70.05)
Specificity (%, 95% *CI*)	92.42 (88.37-95.19)	80.30 (74.88-84.82)	72.73 (66.86-77.92)
PPV (%, 95% *CI*)	93.96 (90.67-96.18)	84.75 (80.39-88.31)	74.74 (69.20-79.59)
NPV (%, 95% *CI*)	93.49 (89.59-96.04)	84.46 (79.25-88.59)	62.54 (56.84-67.92)
C-statistic	0.974 (0.957-0.985)	0.842 (0.810-0.871)	0.688 (0.649-0.725)
*P* value^d^	–	<0.001	<0.001
**Validation Cohort**			
True positive/False negative	104/8	90/22	69/43
False positive/True negative	8/100	27/81	36/72
Sensitivity (%, 95% *CI*)	92.86 (85.98-96.64)	80.36 (71.56-87.03)	61.61 (51.91-70.50)
Specificity (%, 95% *CI*)	92.59 (85.49-96.51)	75.00 (65.58-82.61)	66.67 (56.86-75.27)
PPV (%, 95% *CI*)	92.86 (85.98-96.64)	76.92 (68.05-83.99)	65.71 (55.74-74.52)
NPV (%, 95% *CI*)	92.59 (85.49-96.51)	78.64 (69.24-85.86)	62.61 (53.05-71.31)
C-statistic	0.980 (0.951-0.994)	0.777 (0.716-0.830)	0.641 (0.574-0.705)
*P* value^d^	–	<0.001	<0.001

AT, arrival time; Lesion-lung AT difference, the AT difference between lesion and air-filled lung tissues; PPV, positive predictive value; NPV, negative predictive value.

True positive/False negative: The cases that were definitely diagnosed as malignant were diagnosed as malignant/benign by the methods to be compared.

False positive/True negative: The cases that were definitely diagnosed as benign were diagnosed as malignant/benign by the methods to be compared.

a The prediction model (cutoff value: 44.587%) was obtained for the development cohort and applied to internal and external verification.

b Diagnostic criterion proposed by Bai in 2016: Lesion-lung AT difference ≥ 2.5 s.

c Diagnostic criterion proposed by Caremani in 2008: AT ≥ 10 s.

d R package “compare C” (version 1.31) was used to compare C-statistics of prediction model and the existing US diagnostic criteria.

## Discussion

US has been used in the differential diagnosis of benign and malignant SPLs for decades. In terms of B-US, the lesion shape, internal echo, boundary definition, bronchial inflation sign, relationship with pleura and adjacent organs are valuable (6–8). As for CEUS, the perfusion mode, degree of enhancement, homogeneity, microvascular characteristics, and especially AT are recommended indicators (8–19). However, studies that proposed the above indicators also has limitations, including small sample size, inconsistent conclusions from different researchers, strong subjectivity, and no multi factor analysis (9). In this large-scale and multiparameter study, we constructed a new US prediction model for the differential diagnosis of benign and malignant SPLs and obtained good discrimination and calibration. To our best knowledge, this is the first model based on both B-US and CEUS parameters for diagnosis of SPLs, which can provide radiologists with more accurate diagnostic information than B-US and the existing CEUS diagnostic criteria.

In our model, the time-related quantitative CEUS parameters played a dominant role on the prediction of malignancy probability. Similar to liver, lung also has a dual blood supply comprised of the pulmonary arteries and the bronchial arteries (19, 21, 22). When a malignant tumor develops, the blood supply from the bronchial arteries will markedly increase and gradually replaces the supply from the pulmonary artery, becoming the main source of blood for the tumor (19, 21, 22). And this characteristic can be identified by AT, because the arterial phases of these two types of blood vessels are different (19). It is controversial that different investigators have proposed different cutoff values for AT. Sartori et al. (17) suggested AT > 7.5 s, the arterial phase of a lesion was delayed, indicating a great possibility of malignancy, while Caremani et al. (14) recommended AT > 10 s as the cutoff value. Furthermore, multiple internal and external factors may influence the value of AT (20). Thus, quantitative observations of AT alone as a diagnostic criterion to determine etiology of SPLs is at high risk of misjudgment. To deal with these issues, Bai et al. (13) took the AT of lung tissue as the baseline, calculated the AT difference between of lesion and the baseline, and finally obtained lesion-lung AT difference, which was proven to provide better diagnostic performance.

Our study evaluated both AT and lesion-lung AT difference, and the results showed that their discriminations were inferior to that of our model. We attribute the excellent performance of the model to our newly created parameter, ratio of AT difference, which uses air-filled lung tissues and the thoracic wall as reference, and judges the blood supply source of the lesion by comparing the AT differences of air-filled lung tissues, lesion, and thoracic wall. This parameter represents the ratio of “the time taken by the blood from the pulmonary circulation to the lesion” to “the time taken by the blood from the pulmonary circulation to the systemic circulation”. The smaller the ratio, the more likely the blood supply of the lesion is from the pulmonary circulation. The larger the ratio, the more likely the blood supply of the lesion is from the systemic circulation. This parameter has greater clinical utility because individual differences are excluded to the extent possible. For example, in some patients, ATs of lung, lesion, and thoracic wall are very close, and the lesion-lung AT difference may be far shorter than the cutoff value, so all of these lesions will be classified as benign. But when ratio of AT difference is applied, they can be accurately differentiated because of the invariant proportional relationship.

Different from the above point of view, several studies have shown varying types of arterial supply for some special types of lesions, including mixed arterial supply and even dominant pulmonary arterial supply (22, 23). It seems that it is not rigorous to diagnose only by the source of blood supply. Fittingly, our model also included qualitative CEUS parameters, including vascular sign and non-enhancing region type. For vascular sign, Wang et al. (15) believed that the main features of benign lesions were branching, pointed, patchy and rim-like, while those of malignant lesions were vascular, cotton-like and dead-wood like. However, Caremani et al. (14) described spots, points, and ring-enhancement as the features of malignant lesions, and linear hyperechoic images as the features of benign lesions. These examples clearly demonstrate that complicated subjective classification systems will lead to inconsistent conclusions among researchers. In our study, only distinct tree-like and curly hair-like vascular signs were recorded. The tree-like sign is a feature of the benign lesions, which corresponds to a pulmonary artery with a normal structure, and the curly-hair-like sign is a feature of the malignant lesions, which corresponds to tumor neovascularization with a disordered structure and tortuous form. Compared with the existing studies (14, 15), our classification method is simpler, more specific, and more accurate.

In term of non-enhancing regions, the main feature of malignant lesions is irregular with ragged edges while those of benign lesions are often regular with smooth edges. Tuberculosis, in particular, often presents as a single affected area or as multiple small patches of non-enhancing region early in the course of the disease, which gradually expands and merges into large patches of non-enhancing region as the disease progresses. The non-enhancing region features that we have described are consistent with those of other imaging studies (24–26).

The qualitative parameters in our prediction model are easy to implement with excellent reproducibility, and played an important auxiliary role in the prediction of malignancy probability.

In addition to CEUS parameters, only one B-US parameter, the angle between lesion border and thoracic wall, was included in the final model, which reflects the morphological characteristics of the lesions. In contrast to CT, US cannot easily display the lobes, spinous processes, or burrs on the edge of a lesion because the boundary between the lesion and air-filled lung tissues is not clear in US images (27, 28). However, the boundary between the lesion and thoracic wall is clear and the angle can be easily measured. In particular, pulmonary tuberculosis lesions with hyperplasia as the main feature often showed obtuse angles, which resulted in a large proportion of obtuse angle lesions in the benign lesion group in this study (25, 29). But in general hospitals that are not responsible for tuberculosis diagnosis and treatment, the resulting bias will be minimal.

In addition to the above variables included in the model, some CEUS parameters are also different between benign and malignant lesions, such as perfusion pattern, PI and WOR, which are consistent with previous literatures (8–19). Based on the principle of simplifying the model as much as possible to avoid over fitting, our model only included 6 indicators to synthesize the information of B-US and CEUS. In summary, the method is simple and has strong clinical feasibility.

## Limitations

The primary limitation of this study is that it was conducted at a single center, so there is a possible bias caused by a nonrepresentative distribution of disease types. However, our center is a leading pulmonary hospital. Patients came from all over the country for our study may effectively reduce the bias.

The second limitation is that more features of B-US and color doppler flow imaging were not included in this study, because the gas artifacts and respiratory movement might affect their stability and accuracy. However, these indicators, especially B-US parameters, may still be of certain value in the diagnosis.

Considering that these limitations, we plan to conduct a prospective multicenter study with an increased number of US parameters in order to improve our model further.

## Conclusion

Our model, synthesizing multiple parameters of B-US and CEUS, could contribute to improved performance in the differential diagnosis of benign and malignant SPLs, compared with the existing CEUS diagnostic criteria. It is simple, non-radioactive and has great potential for clinical use.

## Data Availability Statement

The raw data supporting the conclusions of this article will be made available by the authors, without undue reservation.

## Ethics Statement

The studies involving human participants were reviewed and approved by the Institutional Review Board of Shanghai pulmonary hospital (No. K18-197Y). The patients/participants provided their written informed consent to participate in this study.

## Author Contributions

YW, LF and KB had the idea for and designed the study. They had full access to all data in the study and take responsibility for the integrity of the data analysis. KB and YW wrote the first full draft of the report. KB, YZ and YW contributed to image analysis. YZ, M-jS, H-wC, YC, H-mZ, C-hT and JY contributed to critical revision of the report. D-mX and X-fY contributed to the statistical analysis. All authors contributed to the article and approved the submitted version.

## Funding

This study was supported by 2018 Supporting Project of Medical Guidance (Chinese and Western Medicine) of Science and Technology Commission of Shanghai Municipality (18411966700); 2019 Technical Standard Project of Shanghai “Science and Technology Innovation Action Plan” of Science and Technology Commission of Shanghai Municipality (19DZ2203300) and Clinical Research Foundation of ShangHai Pulmonary Hospital (fk1940 and FKLY20015).

## Conflict of Interest

The authors declare that the research was conducted in the absence of any commercial or financial relationships that could be construed as a potential conflict of interest.

## Supplementary Material

Supplementary Video 1 A dynamic ultrasound image of a benign subpleural pulmonary lesion (tuberculosis) in the upper lobe of left lung of a 61-year-old woman. The left half was a B-mode ultrasound image, and the right half was a corresponding contrast-enhanced ultrasound image. “T1” was a timer that was timed from the injection of ultrasound contrast agent. The B-mode ultrasound image showed a wedge-shaped lesion with an acute angle between lesion border and thoracic wall. In the contrast-enhanced ultrasound image, the first enhanced area was air-filled lung tissues (4s), then the lesion (7s), and finally the thoracic wall (14s). It was found that the perfusion pattern was from the hilum to the pleura with a tree-like vascular sign. At 15s, the lesion was completely enhanced, which was heterogeneous and hyper-enhanced, and contained sieve-like non-enhancing regions.

Supplementary Video 2 A dynamic ultrasound image of a malignant subpleural pulmonary lesion (squamous cell carcinoma) in the upper lobe of left lung of a 75-year-old man. The left half was a B-mode ultrasound image, and the right half was a corresponding contrast-enhanced ultrasound image. “T1” was a timer that was timed from the injection of ultrasound contrast agent. The B-mode ultrasound image showed a quasi circular lesion with an obtuse angle between lesion border and thoracic wall. In the contrast-enhanced ultrasound image, the first enhanced area was a main pulmonary artery (8s), then air-filled lung tissues (9s) and the lesion (14s), and finally the thoracic wall (18s). It was found that the perfusion pattern was from the periphery to the center without obvious vascular sign. At 24s, the lesion was completely enhanced, which was heterogeneous and iso-enhanced, and contained an irregular non-enhancing region.
